# Morphological Correlates of Personality in Female Asian Particolored Bats (*Vespertilio sinensis*)

**DOI:** 10.3390/ani10020289

**Published:** 2020-02-12

**Authors:** Yuze Wang, Biye Shi, Xin Zhao, Jiang Feng, Tinglei Jiang

**Affiliations:** 1 Jilin Provincial Key Laboratory of Animal Resource Conservation and Utilization, Northeast Normal University, 2555 Jingyue Street, Changchun 130117, China; wangyz834@nenu.edu.cn (Y.W.); shiby595@nenu.edu.cn (B.S.); 2 Key Laboratory of Vegetation Ecology of Education Ministry, Institute of Grassland Science, Northeast Normal University, 2555 Jingyue Street, Changchun 130117, China; 3 School of Psychology, Inner Mongolia Normal University, Hohhot 010000, China; zhaox111@nenu.edu.cn; 4 College of Life Science, Jilin Agricultural University, 2888 Xincheng Street, Changchun 130118, China

**Keywords:** exploration, activity, aggression, body size, behavioral syndrome

## Abstract

**Simple Summary:**

Personality emerges because of individual differences in repeatable state variables, such as metabolic rate, age, sex, or body size. Personality and its correlation with body size, however, have been relatively unexplored in bats. Our study showed that the exploration of female Asian particolored bats was significantly repeatable, but we did not find significant correlations among exploration, activity, and aggression. This finding suggested that female Asian particolored bats may not have a behavioral syndrome. Additionally, the body mass of female Asian particolored bats was correlated with aggression and activity, suggesting that body mass may have an impact on the behavioral characteristics of bats. Our findings not only added to the literature concerning personality in bats but are also helpful for understanding the maintenance of an animal’s personality.

**Abstract:**

Personality traits represent a leading edge in the evolutionary process, as natural selection acts directly on variations in individual phenotypes within populations. Recent theoretical models have focused on the concept of adaptive state-dependent behavior, proposing that repeatable differences in behavior emerge because of individual differences in repeatable state variables, such as metabolic rate, age, sex, or body size. Personality and its correlation with body size, however, have been relatively unexplored in bats. We used female Asian particolored bats (*Vespertilio sinensis*) to investigate three personality characteristics (exploration, activity, and aggression) using the classic hole-board test and examined their relationships with body size using an information-theoretical approach. Our results showed that the exploration of female Asian particolored bats was significantly repeatable, but we did not find significant correlations among the three personality traits. This finding suggested that the female Asian particolored bat may not have a behavioral syndrome. In addition, the body mass of female Asian particolored bats was positively correlated with aggression but was negatively correlated with activity, suggesting that body mass was an important physiological basis affecting the behavioral characteristics of female Asian particolored bats.

## 1. Introduction

Personality is defined as the consistency of an individual’s behavioral responses over time and across situations [1,2]. Exploration, boldness, activity, and aggression may be the most commonly measured personality traits [3]. The causes for animals to have personalities, however, remain poorly understood [4]. The state-dependent behavior model emphasizes that the behavior of individuals is affected by different states, and these state variables are “fixed” to a large extent, leading to the repeatability of behaviors [5,6,7]. State typically refers to any intrinsic morphological or physiological characteristic of an individual (e.g., age, sex, metabolic rate, immune state) or its information state (e.g., skill set, social rank, social role) that affects the costs and benefits of behavioral decisions [8,9]. Body size is an important aspect of an individual’s intrinsic state that can directly affect fitness-related traits as well as the costs and benefits associated with behavioral decisions [10,11,12,13,14]. As such, body size differences could set the stage for positive feedback loops between state and behavior that shape animal personality during development along various behavioral axes [9]. For example, one study found that the larger hatchling keelback snakes (*Tropidonophis mairii*) exited from shelter more quickly and more boldly [11]. Another study found that differences in size in laboratory rats (*Rattus norvegicus*) were significantly correlated with differences in exploratory behavior, with larger individuals being more exploratory [15]. To date, two hypotheses have been developed to explain the relationships between body size and personality traits. The metabolic hypothesis states that smaller individuals have lower fat reserves and a higher metabolic rate, so they are forced to forage more actively than larger individuals [16,17,18]. For example, the metabolic rate of great tits (*Parus major*) was positively correlated with risk-taking behavior [19]. A negative correlation, however, was observed between the metabolic rate and exploratory behavior in female great tits [20]. The predation hypothesis states that smaller individuals are more vulnerable to predation than larger individuals, so they are more vigilant than large individuals in a new environment [21]. Previous studies have shown an inconsistent relationship between body size and personality traits within and across species, suggesting a complex mechanism underlying the relationships. In this case, the relationships between individual differences in body size and personality should be investigated in a wider variety of taxa.

A syndrome is defined as a suite of correlated behaviors reflecting consistent individual differences across multiple situations [2,22,23]. Behavioral syndromes allow individuals to be characterized on primary axes summarizing several personality traits; for example, the proactive-reactive axis [22,24]. Proactive individuals tend to be more active and aggressive than their conspecifics and show rapid, superficial exploration of a novel environment [24]. Recently, several theoretical models exploring the concept of state dependency have been used to explain the proximate causes of animal personality and behavioral syndromes [5,6,7,25]. Individual differences in state are reflected in individual differences in behavior. Moreover, in different environments, different types of personality traits associated with the same state may be correlated [7].

To date, personality has been studied in a number of animal groups, including fish [26], birds [27,28,29], mice [30,31], and primates [32,33]. The alternate selection pressures imposed by environmental differences among species have suggested that behavior patterns and their relationships may be unpredictable within and across species [34]. Therefore, it is necessary to explore personality variation in a wider variety of wild animals. Bats are the second-largest group of mammals. The ecological and morphological diversity of bats facilitates the study of cross-species personality variation in phylogenetic and environmental settings [35]. Similar to nonhuman primates [36], bats represent a potentially valuable taxon for studies of personality variation within species [35]. Studies on the personality of bats are rare, however. For example, the activity of little brown bats (*Myotis lucifugus*) is influenced by age [35], and ectoparasite prevalence and intensity are influenced by the activity of little brown bats [37]. The Spix’s disc-winged bat (*Thyroptera tricolor*) within social groups exhibits consistent individual differences in vocal behavior [38,39]. The isolated behaviors of big brown bats (*Eptesicus fuscus*) are not necessarily correlated with socially oriented behaviors [40,41,42]. These previous studies confirmed that bats have distinct personality traits, but the relationships between personality and body size remain unclear. Thus, more research on personality and its causes is needed.

Asian particolored bats (*Vespertilio sinensis*) often cluster as groups within natural or artificial cavities, suggesting a highly complex social structure [43,44]. Outside the mating season, males and females separate [45,46]. Our previous studies have shown that Asian particolored bats frequently compete with each other for a more central roosting spot [47,48]. Competition among females is more intense because of the energy needed during gestation [49]. The social niche specialization hypothesis states that species considered more social should have more distinct personality differences [50]. Thus, we predicted that Asian particolored bats would have distinct personality traits (exploration, activity, and aggression) and behavioral syndromes. Additionally, body size can directly affect personality traits [9], and larger individuals are more exploratory [15], aggressive [19], and active [9]. We thus predicted that we would detect significant relationships between exploration and body size, between aggression and body size, and between activity and body size.

## 2. Methods

### 2.1. Bat Capture and Husbandry

We collected 52 female adult bats from a highway bridge (126°57′26′′ E, 45°32′52′′ N) in Acheng, Heilongjiang Province, China. Because of the separation of males and females in the breeding season, this colony at that time was a nursery of female Asian particolored bats. In this study, we selected only adult females (two-year-old individuals) for the experiments based on our previous banding data. We collected nonlactating females using mist nets in June, July, and August. The sample sizes at the three trapping times were 9, 23, and 20. We determined female reproductive status by examining the nipples and by palpation of the abdomen [51]. We placed the bats into cloth bags and brought them to a field temporary feeding room (5 × 6 × 2 m). The temporary feeding room was quiet and consistent with the natural light cycle. Temperature and humidity of the room were similar to the bats’ natural habitat (temperature: 18–23 °C; relative humidity: 60%–80%).

### 2.2. Hole-Board Test

**Figure 1:**
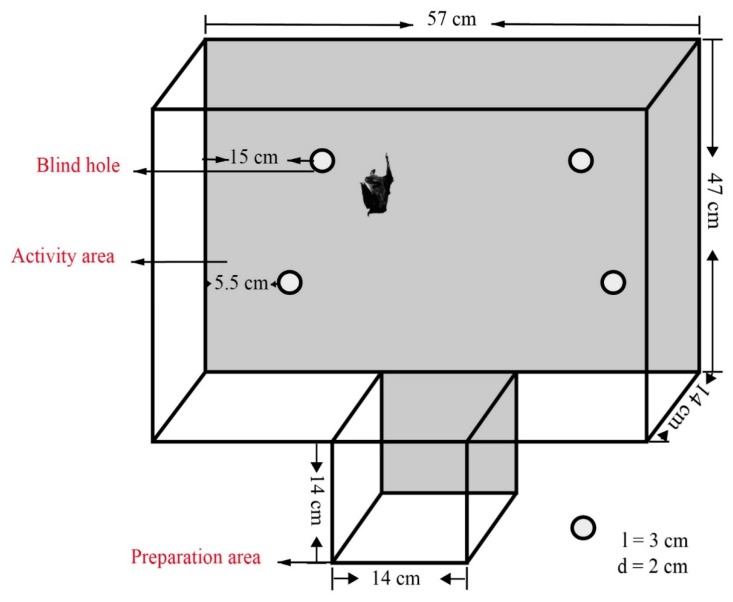
Experimental setup for the hole-board test: The hole board consists of the activity area (57 × 42 × 14 cm) and the preparation area (14 × 14 × 14 cm) separated by sliding doors. Four blind holes are located on the inner surface for bats to explore. The shaded area is the area covered by the gauze.

Because Asian particolored bats are good at climbing and like to perch in crevices, we used a hole board to examine two personality traits: exploration and activity. The hole board consisted of an activity area (57 × 42 × 14 cm) and a preparation area (14 × 14 × 14 cm) at the bottom, and the two areas were separated by sliding doors (Figure 1). The surface of the device had a transparent cover, and the surface of the activity area was covered with gauze for the bats to climb. We drilled four blind holes (3 cm in diameter and 2 cm in depth) on this inner surface. Two holes were located near the center of the chamber (15 cm from the nearest wall), and the other holes were located near the corner (5.5 cm from the nearest wall). The hole-board test quantified the activity and exploration traits of bats. For example, the greater the number of times the bat explored the holes, the more exploratory it was considered. This also included exploring the middle hole more frequently than the edge hole [35]. The longer time the bat spent moving toward the hole board and the shorter time it exhibited latency to enter, the more active it was considered.

During the experimental periods, we gently moved the bats from the feeding room to the lab in a cloth bag (all under the same conditions as in the feeding room). When the behavioral recording began, we placed each bat in a separate preparation area for two minutes to acclimatize. If a bat did not enter the activity area within one minute, we nudged it gently into the activity area. We then closed the sliding door to prevent the bat from returning. We used an infrared camera (Sony Handycam HDR-CX760E, Tokyo, Japan) to record the behavior of bat within 10 min after entering the activity area. We conducted all of the experiments at night to match the bats’ circadian rhythms. After the completion of an individual trial, we cleaned the hole board with alcohol to eliminate the odor of the previous bat, and the next bat’s experiment was conducted after all the alcohol had evaporated. After the hole-board test, we trained the bats to feed on their own using *Tenebrio molitor* and water, and then we transferred the bats to separate cages (bats could communicate vocally, but not interact physically). To decrease the effects of repeated tests on stress accumulation in the Asian particolored bats [31], we repeated the hole-board test for each individual after the aggression test was finished. We carried out the hole board test repeatedly with different bats in June, July, and August.

### 2.3. Aggression Test

Réale et al. [2] defined animal aggression as an individual’s agonistic reaction toward conspecifics. The more aggressive an individual is, the more times it takes the initiative to attack [52]. After the bats were able to feed by themselves, we randomly selected six bats by drawing lots to form groups to test for aggression. In this study, we selected a total of six groups for the experiments. No bats belonged to more than one group. Each group was raised in a cage (50 × 50 × 50 cm) in a feeding room during the experimental period. We marked bats with 5.2 mm aluminum alloy bands (0.05 g, which is less than 0.25% of the bat’s body mass; Porzana Ltd., Icklesham, U.K.). Our previous studies on Asian particolored bats showed that the tag ring did not harm the bat or interfere with its behavior [47,48]. Aggression occurs when individuals compete over limited resources [53]. Asian particolored bats display aggressive behavior as intruder bats compete for a more central roost position by pushing with their forearms and heads over resident bats [48]. We thus defined aggression as one bat using its head or forearms to push against other bats. In this case, we recorded the aggressive behavior of each group using an infrared camera (Sony Handycam HDR-CX760E). We made these recordings from 22:00 to 07:00 the next day.

### 2.4. Morphological Measurements

After the first hole-board test, we measured the bats’ body mass (BM), forearm length (FAL), and head body length (HBL). We measured BM using an Ohaus LS 200 electronic scale (Ohaus, Parsippany, NJ, USA) with an accuracy of 0.01 g. We measured FAL and HBL using digital display calipers (TESA-CAL IP67, 0.01 mm, Renans, Switzerland). We measured all body size parameters and used the average value of the subsequent statistical analysis.

### 2.5. Personality Traits Analysis

We used a Solomon Coder (András, 2012) to analyze videos recorded from the hole board test. First, we created a Configuration file in the Solomon Coder, which contained the behavioral elements created by the user, their categories, and the layout of the user interface. On the user interface, each behavioral element was represented as a button. For an array of videos taken in the same experimental setup, the same configuration file could be used. After opening the video under the Configuration file and setting the start time, we could click a behavior button according to the corresponding behavior. The data were stored in a table (coding sheet) and then were exported in the form of arch. We saved and exported for later statistical analysis.

We used eight behaviors in the Solomon Coder to quantify Asian particolored bats’ personalities according to the previous studies [31,35]:

(1) Latency to enter: the amount of time it takes to enter the activity area from the preparation area.
(2) Frequency of head dips: the number of times bats explored the hole.
(3) Latency to head dip in holes (edge): the time it takes from entering the active area to exploring the edge hole for the first time.
(4) Latency to head hip in holes (center): the time it takes from entering the activity area to exploring the central hole for the first time.
(5) Locomotion: the proportion of time spent climbing and crawling in relation to the total duration of the experiment in the activity area.
(6) Echolocation: the proportion of time spent echolocating in relation to the total duration of the experiment in the activity area.
(7) Grooming: the proportion of time spent grooming in relation to the total duration of the experiment in the activity area.
(8) Urination and defecation: the total number of urination and defecation events.

In this study, we used a QvodPlayer with a resolution of 25 frames/s (Version 5.0.80, Shenzhen Qvod Technology Co., Ltd., Guangdong, China) to perform a frame-by-frame video analysis of aggressive behavior and to count the attacks between all individuals during at least one week. We defined an attack as an intruder bat successfully occupying the central roost position or giving up pushing. It is common to measure aggression in terms of frequency or proportion. For example, in studies of big brown bats (*E. fuscus*), the frequency of biting was used as a measure of aggression [40]. The aggressiveness of Wildtype Groningen rats (*Rattus norvegicus*) was measured by the proportion of the time spent on aggression to the total experimental time [30]. Thus, here, we recorded the number of times that an individual bat actively attacked other bats and calculated the ratios of an individual bat’s attack times to the total number of attacks in a group. We used the ratios to quantify the individual bat’s aggression. The higher the proportion of attacks an individual bat initiated in its group, the more aggressive we considered it to be.

### 2.6. Statistical Analysis

We performed a principal component analysis (PCA) on the eight behavioral variables using the psych package [54] and GPArotation package [31,55] in R 3.5.1 [56]. We dimensionalized eight kinds of behavior in the two hole-board experiments, and used the Kaiser-Guttman criterion was used to select the number of major components retained [57]. We then used each retained principal component score (PCS) as a composite behavioral variable for subsequent statistical analysis. We conducted intra-species repeatability tests for PCS using a one-way analysis of variance (ANOVA) in SPSS (IBM Corporation, Armonk, NY, USA) and obtained intra-class correlation coefficients (ICC) from the analysis. ICC results are available only when the *P* value of ANOVA results is greater than 0.05. Landis and Koch [58] suggested that the larger the ICC value, the higher the repeatability of personality. For example, 0.80 is very stable, 0.61–0.80 is medium, 0.41–0.60 is normal, 0.11–0.40 is low, and less than 0.1 is inconsistent. In this study, we tested only the repeatability of exploration and activity, because the data for aggression were not suitable for analysis. Animal competition may lead to the formation of dominance hierarchies, which leads some individuals to avoid competition [59]. We also found that female Asian particolored bats formed near-linear dominance hierarchies [60]. Thus, it may not be feasible to test the repeatability of aggression. To verify whether the behavioral syndromes in female Asian particolored bats existed, we first used Kolmogorov–Smirnov (K-S) tests to assess normality of the three personality scores (exploration, activity, and aggression). Because the data were normally distributed (*p* > 0.05), we performed Pearson correlations to assess the relationship between activity and exploratory scores, between aggression and activity scores, and between aggression and exploratory scores.

We calculated the variance inflation factor (VIF) of the predictor variables (FAL, HBL, BM) to determine whether a multiple linear model was applicable. We found that VIF was <5 for relationships between any two variables, which suggested that a multiple linear regression could be used to test the relationships between personality and body size [61]. To test the relationships between personality and body size, we selected optimized linear models using the lmer function in the package “lme4” [62] in R 3.5.1 [56]. In the models, we used the personality traits (activity or exploration) as dependent variables, the body sizes (FAL, HBL, BM) as independent variables, and date of capture (month) as a random factor. Additionally, in the model to determine drivers of aggression, we used aggression as the dependent variable, the body sizes (FAL, HBL, BM) as independent variables, and month and group as the random factors. The models generated a set of candidate models, including the main effects of the independent variables and all possible combinations of these main effects. We compared the competing models with the corrected Akaike information criterion (AICc) for small sample sizes [63]. We calculated the differences between AICc values as follows: Δ*i* = AIC*i* – AIC*min*. Additionally, because ΔAIC > 2 is considered to be the gold standard for model selection [64], we performed multimodel inference if differences in AICc were ≤ 2, using the model.avg function in the package “MuMIn” [65] in R 3.5.1 [56]. We also calculated Akaike weights (*wi*) to explain the relative likelihood of a given model; these values stood for the normalization of the probabilities of the different models given the data [66]. We also used the K-S test to test the normality of the residuals of the best models. Additionally, the BM of an individual bat was 25.35 g, which was much heavier than the other bats. We did not consider this value of the individual bat to be an outlier because bats with similar BM are common in the population based on our long observation.

### 2.7. Ethical Statements

We adhered to the Association for the Study of Animal Behavior/Animal Behavior Society Guidelines for the use of animals in research. The procedures in this study were observational and noninvasive. The Wildlife Conservation Office of the Jilin Forestry Department, China, approved this study (approval number: NENU-W-2014-101). We conducted bat capture methods and experiments within the regulations set by the Northeast Normal University. After the experiments, we returned all bats to the site of capture. Each bat spent less than a month in the lab, and no bats died or were injured during the experiment.

## 3. Results

### 3.1. Characteristics of Personality

The first three principal components explained 70% of the total variance. The first principal component was related to exploratory behavior, including frequency of head dips, latency to head dip in holes (edge), latency to head dip in holes (center), and echolocation. The lower the score of the first principal component, the stronger the exploratory ability. The second principal component reflected a series of activities related to overall activity level, including latency to enter, locomotion, and echolocation. The lower the second principal component score, the higher the activity level. The third principal component was related primarily to grooming and excretion (Table 1). The total numbers of attacks in the six groups of Asian particolored bats were 171, 214, 89, 123, 242, and 175. We analyzed aggression from 36 individual bats with an average of 0.167 per bat (SD = 0.101).

The ICC results showed that the first principal component had significant consistency (ICC = 0.361, *df* = 51, *p* = 0.004), suggesting that exploration was repeatable. Exploration was not highly stable, however, because the ICC value was relatively low. We did not find significant repeatability in the second (ICC = 0.181, *df* = 51, *p* = 0.101) or third (ICC = −0.029, *df* = 51, *p* = 0.579) principal components between two hole-board tests.

### 3.2. The Relationships Among Different Personalities

In this study, all three personality scores were normally distributed (exploration: *N* = 52, *p* = 0.075; activity: *N* = 52, *p* = 0.200; aggression: *N* = 36, *p* = 0.200). We obtained the exploratory score (the first principal component) and activity score (the second principal component) by PCA. We did not identify a significant relationship between exploratory and activity scores (*r* = −0.40, *p* = 0.816). There were no significant correlations between aggression and activity (*r* = 0.263, *p* = 0.122) or between aggression and exploration (*r* = −1.16, *p* = 0.502).

### 3.3. The Relationships Between Personality and Body Size

**Figure 2:**
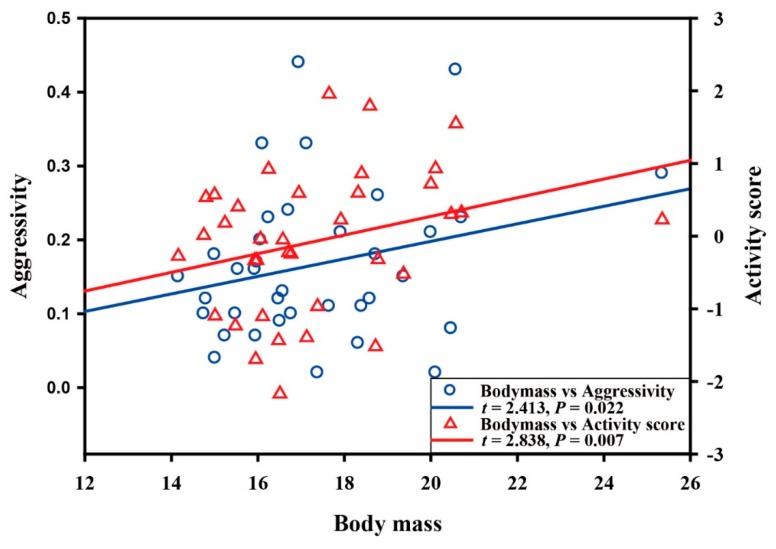
Relationships between body mass and activity score (red), and between body mass and aggression (blue). Here, the activity score was negatively correlated with activity. The *t* and *P* values were obtained from the best models (see Table 2 and Table 4).

Our results indicated that the best model of the relationship between activity and body size in Asian particolored bat used BM as the predictor variable (Table 2). Bats with large activity scores were associated with heavier BM (*t* = 2.838, *p* = 0.007; Figure 2). Moreover, in the averaged model, BM had a significant effect on activity score (Table 3), suggesting that it was appropriate to use the first model as the best model. In this study, the lower the second principal component scores, the higher the activity. In this case, the heavier BM, the lower the activity in Asian particolored bat. Additionally, we found that the residuals of the best model conformed to normal distribution (*p =* 0.755).

In the model selection process for aggression, we obtained only one model (Table 4), and this model used BM and HBL as the predictor variables. Bats with higher aggression were associated significantly with heavier BM (*t* = 2.413, *p* = 0.022; Figure 2), but the relationship between aggression and HBL was not significant (*t* = −1.923, *p* = 0.063). In this case, the heavier the BM, the higher the aggression score. Additionally, we found that the residuals of the best model conformed to normal distribution (*p =* 0.649)

In the model selection process for exploration, we obtained only one model, and this model used HBL as the predictor variable. Simple linear regression, however, showed that there were no significant correlations between exploratory score and head body length (*t* = 1.156, *p* = 0.253). We also found that the residuals of the best model conformed to normal distribution (*p =* 0.132).

## 4. Discussion

In this study, we examined the personality of Asian particolored bats through the classic hole-board test, and we used an information-theoretic approach to estimate the effects of body size on the bats’ personalities. The results showed that Asian particolored bats demonstrated behavioral repeatability, especially regarding exploration, but we did not identify a behavioral syndrome present in this species. These results partially supported our first hypothesis. Additionally, we found that body size influenced activity and aggression rather than exploration, supporting our second hypothesis.

### 4.1. Characteristics of Personalities

Similar to the hole-board tests employed for little brown bats [35], we successfully used the hole-board device to separate the exploration and activity of Asian particolored bats to some extent. The first principal component from the PCA expressed the extent of the individual bat’s exploration. The length of the delay in exploring the holes may indicate fear of venturing forth and leaving the walls around the test area [35]. A shorter delay may indicate a bolder individual bat, which may have similar motivation to explore [67,68]. The second principal component was related to latency to enter, locomotion, and echolocation. In rodents, latency to enter has been considered as a measure of emotionality or a measure of the activity of the sympathetic nervous system in response to stressful stimuli [31,69,70]. Therefore, we suggested that latency to enter might express the activity level of Asian particolored bats under the pressure of a new environment. The third principal component was related to grooming and excretion. Sudden grooming behavior in rodents is a component of the stress response, indicating increased levels of anxiety [31]. Excretion is often thought of as an activity that measures the emotional or sympathetic nervous system’s response to stressful stimuli [31,69]. Additionally, novelty may elicit exploratory behavior as well as neophobic reactions [71]. Moreover, high levels of fear may lead to neophobia and low levels of exploration [72]. Thus, grooming can be evoked when animals encounter exotica or feel stressed or fearful [73,74]. Therefore, the third principal component may express the anxiety and neural response of Asian particolored bats when they faced a novel environment. Further studies will need to confirm such speculation.

Exploratory repeatability ensures that the variation observed at the phenotypic level is not caused solely by microenvironments at the time of measurement. Many studies have estimated repeatability as a first step toward studying the genetic basis of a behavior [75]. Repeatability may reflect the potential heritability of exploratory behavior [31,76], but some studies have not supported this view [77]. Additionally, a large number of studies have quantified differences in how animals explore new environments through non-field tests, hole-board tests, and new object tests and have found that the intensity of exploration decreased with the number of times that animals were habituated to non-field tests and new object tests [2,78,79,80,81]. In this case, habituation can be used to explain why exploration in the Asian particolored bat is not highly stable. Our results were credible because exploration commonly decreases with the number of experimental times in studies of personality.

Contrary to exploration, activity in Asian particolored bats was not repeatable. There were two possible reasons. First, behavioral plasticity allows organisms to adapt to the surrounding environment [82,83]. Because of the differences in sensitivity among individuals, the degree of change in personality will differ [35]. In our study, the activity of Asian particolored bats was not repetitive, possibly because of the differences in individuals’ sensitivity to new environments (long personal observation by Yuze Wang). Second, Alison et al. [75] found that animal activity is inherently less repetitive than other behaviors. Moreover, some studies have found that animal activity is not repetitive [84,85]. Thus, it may be reasonable that bats’ activity did not show repeatability. Further experimental investigation in additional bat taxa is required to clarify the repeatability of activity.

### 4.2. The Relationships Among Different Personalities

Personality traits in species from a variety of taxa are often, but not universally, correlated [86,87,88]; but see several previous studies [72,89]. The intensity of behavioral relevance and the frequency of occurrence of different behavioral types, however, may vary between populations [89,90] and among social contexts [26,91,92,93]. For example, the solitary and socially directed behaviors of individual big brown bats (*E. fuscus*) are not necessarily relevant [40]. In this study, we tested aggression when Asian particolored bats competed with conspecifics for roosting space, but we measured activity and exploration in isolated bats. In this case, it seems reasonable that we did not observe any significant relationships between aggression and activity, or between aggression and exploration.

Similar to our study on Asian particolored bats, absence of correlation between personality traits is also common. For example, the exploration of European house crickets (*Acheta domesticus*) was positively correlated in anti-predation situations, but no correlation was observed between aggression and activity [94]. There were no significant correlations between exploratory and neophobia in birds, such as black-capped chickadees (*Poecile atricapillus*) [27], mountain chickadees (*Poecile gambeli*) [29], and blue tits (*Cyanistes caeruleus*) [88]. Thus, confirmation of behavioral syndromes requires more species, more diverse personality traits, and more contexts. In this case, only further experimental examination in additional bat taxa to confirm behavioral syndromes.

### 4.3. Effects of Body Size on Personalities

Personality traits may reflect individual differences in patterns of potential physiological mechanisms [95]. In this study, activity was negatively correlated with BM. The influence of BM on the activity of Asian particolored bats may be based on the internal differences in metabolic rate among individual bats with different BMs. BM is influenced both by structural size and the amount of energetic reserves [96]. In general, smaller bats have a higher metabolic rate than larger bats, and therefore, they are considered to have a higher proportion of energy requirements [97,98,99,100]. Because of the higher energy costs, smaller bats have to spend more time foraging [10,18,101,102], which means that they have higher activity levels than larger bats. Our findings were consistent with predictions of the metabolic hypothesis of animal personality [16,17,18].

Normally, larger individuals in most species have a stronger competitive ability, so it is common for larger individuals to attack smaller ones [103,104,105]. In this study, we found that aggression was significantly and positively associated with BM in Asian particolored bats. Additionally, we also found that body size was significantly positively correlated with dominance rank in Asian particolored bats [60]. Larger individuals occupied a more central position in the day roost, and thus effectively reduced heat loss [106,107]. In contrast, smaller bats with weaker competitive ability occupied a marginal area of the roost. In this case, they had to spend more energy to maintain body temperature, thus leading to lower aggression.

The predation hypothesis states that smaller individuals are more vulnerable to predation than larger individuals, so they are more vigilant (lesser exploration) than large individuals in a new environment [21]. In this study, we did not observe a significant correlation between body size and exploratory behavior in Asian particolored bats, similar to the results of previous studies. For example, exploratory abilities of black-capped chickadees (*Poecile atricapillus*) and female zebra finches (*Taeniopygia guttata*) were independent of physical condition [27,108]. Therefore, the relationship between body size and exploration/boldness in bats needs to be explored in more environments and in more species.

## 5. Conclusions

In this study, we used the classic hole-board test to investigate three personality characteristics (exploration, activity, and aggression) in female Asian particolored bats, and we tested the effects of body size on their personalities using an information-theoretic approach. We found that the exploratory behavior of female Asian particolored bats was significantly repeatable, but we did not observe significant relationships among personality traits, suggesting an absence of behavioral syndrome. Moreover, the BM of female Asian particolored bats was significantly correlated with aggression and activity, which showed that body mass was an important physiological factor affecting personalities in female Asian particolored bats.

## Figures and Tables

**Table 1:** Summary of principal component analysis of the hole-board test data for 52 female Asian particolored bats.

Variables	Components		
	PC1	PC2	PC3
Latency to enter	−0.26	**0.77**	0.06
Frequency of head dips	**−0.92**	−0.08	−0.02
Latency to head dip in holes (edge)	**0.78**	0.24	0.07
Latency to head dip in holes (center)	**0.77**	−012	−0.05
Locomotion	**−0.58**	**−0.63**	−0.21
Echolocation	0.35	**0.72**	−0.21
Grooming	0.31	0.03	**0.82**
Urination and defecation	**−0.43**	−0.06	**0.60**
Standard deviance (% of total variance)	0.36	0.20	0.14
Cumulative proportion of total variance	0.36	0.56	0.70

Note: PC1, first principal component score; PC2, second principal component score; PC3, third principal component score. Components were retained for further analysis (using Kaisere-Guttman criterion) and coefficients larger than 0.4 in absolute value (in retained components only) were in bold.

**Table 2:** Candidate linear mixed models explaining the variation in activity of female Asian particolored bats based on body size and date of capture as a random factor. The initial full model was of the form activity ~BM + FAL + HBL + (1 | Month).

Model	Predictive Variables	df	LogL	AICc	Δ_i_	w_i_
1	BM (+)	4	−69.398	147.6	0.00	0.461
2	HBL (−), BM (+)	5	−68.739	148.6	1.14	0.261
3	FAL (+), BM (+)	5	−69.354	150.0	2.37	0.141
4	FAL (+), HBL (−), BM (+)	6	−68.673	151.2	3.57	0.078
5	Intercept	3	−73.280	153.1	5.41	0.031

Note: Models are ranked by Akaike’s information criterion corrected for small sample sizes (AICc) values, from the best to the worst model. The sign of the coefficient of the relationships between activity and body size is shown in parentheses (+, positive; −, negative). In the model, activity was considered to be a response variable; body size (FAL, HBL, BM) were included as fixed factors, and dates of capture was included as a random factor. LogL, Loglikelihoods; Δi, difference between the AICc of each model and the AICc of the best model; *wi*, Akaike weights; FA, forearm length, body mass; HBL, head body length; Month, date of capture.

**Table 3:** Model-averaged parameter estimates of the best-supported (before and including the null model) linear mixed models describing activity variation with body size in female Asian particolored bats.

	Estimate	SE	Adjusted SE	z	95% CI
(Intercept)	−2.425	2.415	2.465	0.984	(−7.263, 2.241)
BM	0.181	0.063	0.065	2.798	(**0.054**, **0.308**)
HBL	−0.043	0.040	0.041	1.049	(−0.124, 0.033)
FAL	0.025	0.069	0.071	0.351	(−0.115, 0.159)

Note: SE, Standard Error; CI, confidence intervals; Parameters with 95% confidence intervals that did not overlap zero are highlighted in bold. FAL, forearm length; BM, body mass; HBL, head body length; Month, date of capture.

**Table 4:** Candidate linear mixed models explaining variation in aggression in female Asian particolored bats based on body size, date of capture, and group as a random factor. The initial full model was of the form aggression ~BM + FAL + HBL + (1 | Month) + (1 | Group).

Model	Predictive Variables	df	LogL	AICc	Δ_i_	w_i_
1	HBL (−), BM (+)	6	34.472	−54.0	0.00	0.303
2	Intercept	4	31.281	−53.3	0.78	0.206

Note: Models are ranked by Akaike’s information criterion corrected for small sample sizes (AICc) values, from the best to the worst model. The sign of the coefficient of the relationships between aggression and body size and date of capture is shown in parentheses (+, positive; −, negative). In the model, aggression was considered a response variable. Body sizes (FAL, HBL, BM) were included as fixed factors, and date of capture (Month) and group were included as random factors. LogL, Log likelihoods; Δi, difference between the AICc of each model and the AICc of the best model; *wi*, Akaike weights; FAL, forearm length; BM, body mass; HBL, head body length.

## Data Availability

The raw data has been upload as supplementary information.

## Supplementary Materials

Supplementary File 1
